# Surgical effect and prognostic factors of myasthenia gravis with thymomas

**DOI:** 10.1111/1759-7714.13396

**Published:** 2020-03-19

**Authors:** Wenxin Tian, Xiao Li, Hongfeng Tong, Wenhan Weng, Fan Yang, Guanchao Jiang, Jun Wang

**Affiliations:** ^1^ Department of Thoracic Surgery Peking University People's Hospital Beijing China; ^2^ Department of Thoracic Surgery Beijing Hospital, National Center of Gerontology; Institute of Geriatric Medicine, Chinese Academy of Medical Sciences Beijing China

**Keywords:** Myasthenia gravis, prognosis, thymectomy, thymoma

## Abstract

**Background:**

To evaluate the surgical effect and prognostic factors of extended thymectomy for myasthenia gravis (MG) patients with thymomas.

**Methods:**

Patients with MG with thymomas who underwent extended thymectomy at Peking University People's Hospital and Beijing Hospital between January 2010 and December 2018 were retrospectively enrolled. Patients were followed up by telephone or outpatient record review . Statistical analyses were performed using SPSS version 19.0.

**Results:**

A total of 194 patients were included in this study. According to the Osserman classification, there were 56 type I, 52 type IIa, 67 type IIb, 14 type III, and five type IV. Video‐assisted thoracoscopic surgery (VATS) thymectomies were performed in 137 patients, and transthymectomies in 57 patients. The average operation time was 136.6 ± 46.5 minutes, average blood loss was 129.3 ± 287.4 mL, and average postoperative stay was 8.3 ± 7.4 days. A total of 170 patients (87.6%) were successfully followed up. The median follow‐up period was 45 months, and the five‐year overall survival (OS) rate was 81.9%. Cox regression analysis demonstrated that age, Masaoka stage, and recurrence were prognostic factors of OS. Tumor recurrence tended to occur in patients with Masaoka stage III + IV, and age was a protective factor. A total of 20 patients experienced postoperative myasthenic crisis (POMC). Univariate analysis indicated that presence of bulbar symptoms, surgical procedure, and blood loss were risk factors for POMC, but multivariate analysis only indicated the presence of bulbar symptoms as an independent risk factor. A total of 162 patients were evaluated for post intervention MG status. A total of 55 patients achieved complete stable remission; the overall effective rate was 84.5%. Older patients and those with B‐type thymomas had a lower probability of achieving complete stable remission. Efficacy was similar in patients who underwent VATS or the transsternal procedure.

**Conclusions:**

Age, Masaoka stage, and recurrence were prognostic factors of OS. Presence of bulbar symptoms was an independent risk factor for POMC. Age and World Health Organization classification influence the postoperative effect of MG.

**Key points:**

**Significant findings of the study**

Age, Masaoka stage, and recurrence were prognostic factors of OS for MG with thymomas. The presence of bulbar symptoms was an independent risk factor for POMC. Age and World Health Organization classification may influence the postoperative effect of MG.

**What this study adds**

Our study had a relatively large sample size of MG patients with thymomas only. We emphasize the analysis of the postoperative effect of MG and overall survival for these patients, which is a complement to previous studies.

## Introduction

Thymomas are the most common thymic epithelial tumors located in the anterior mediastinum. An estimated 20%–40% of patients with thymomas have paraneoplastic syndromes, of which the most common is myasthenia gravis (MG), and around one‐third of thymomas are complicating MG. MG is an autoimmune disease characterized by a neuromuscular junction disorder leading to fluctuating weakness of the skeletal muscles. Thymomas occur in approximately 15% of MG patients. Definitive treatment of thymoma is indicated for all patients with MG regardless of antibody status and MG type. Surgical resection of thymoma in patients with MG consists of complete resection of the thymus and the surrounding anterior mediastinal fat tissue. Surgical approaches include open transsternal and minimally invasive procedures. Less invasive approaches are reportedly associated with less morbidity and faster recovery. However, all studies to date have a small sample size and rarely evaluated detailed post intervention MG status. To evaluate the surgical effect and prognostic factors of extended thymectomy for MG patients with thymoma, we reviewed 194 MG patients with thymoma from the Department of Thoracic Surgery at Peking University People's Hospital and Beijing Hospital between January 2010 and December 2018.

## Methods

### Patients

This study was approved by the Institutional Ethics Review Board of Peking University People's Hospital and Beijing Hospital. Informed consent was waived due to the retrospective nature of the study.

MG patients with thymoma who underwent surgical treatment at Peking University People's Hospital and Beijing Hospital between January 2010 and December 2018 were retrospectively collected. A total of 194 patients (99 men, 95 women) were included. The median age was 49.9 years (range: 15–83 years). Clinical and pathological information, including perioperative data, World Health Organization (WHO) classification, and modified Masaoka staging, was collected. A diagnosis of MG was confirmed through clinical presentation and electrodiagnostic tests such as repeated nerve stimulation with low and high frequencies and simple‐fiber electromyography. MG clinical severity was graded by Osserman classification.

### Surgical procedure

All patients underwent extended thymectomy by unilateral video‐assisted thoracoscopic surgery (VATS) or the transsternal open approach. Procedure was chosen based on tumor characteristics and surgeon preference. Adjacent lymph nodes were routinely removed. The partial pericardium and lung were resected if invaded by the tumor, and any dissemination metastasis was also resected for Masaoka IVa patients.

#### VATS thymectomy

Patients were placed in a hemi‐decubitus position with the ipsilateral upper extremity flexed and fixed onto the head shelf under general anesthesia. A double‐ or single‐lumen tube with a blocker was used for intubation. Right lateral VATS was mainly adopted, and when the tumor was located on the left side of the mediastinum, left lateral VATS was chosen. The side of the chest of surgery was tilted upward by 30°. Surgery was generally performed using a three‐port technique. An observation port was made at the fifth intercostal space of the midaxillary line. Two utility ports were made separately at the third intercostal space of the anterior axillary line and the fifth intercostal space of the midclavicular line; sometimes the third intercostal port was omitted. Artificial pneumothorax can be used according to surgeon preference. The thymoma, whole thymus, and all surrounding mediastinal fat tissues were resected.

#### Transsternal thymectomy

Patients were placed in the supine position with the shoulders upward under general anesthesia. A single‐lumen tube was used for intubation. Through a complete sternotomy, the sternum was retracted using a retractor. After entering the mediastinum, the thymoma, whole thymus, and all associated fat tissues were resected.

### Postoperative treatment

After surgery, all patients resumed their preoperative medications to control the MG. Oxygen saturation and electrocardiographic parameters were monitored during the early postoperative period. A chest radiograph was obtained on the first or second day postoperatively. The chest tube was removed if the drainage had been less than 200 mL in the previous 24 hours. Patients were advised to continue outpatient or hospitalization treatment for MG in the Neurology Department after discharge from the Thoracic Surgery Department. Postoperative radiotherapy was administered to patients with locally advanced tumors (Masaoka stage II–IV) or incompletely resected tumors (R1/R2 resection). Postoperative chemotherapy was suggested for patients with Masaoka stage IV or R2 resection.

### Follow‐up

Follow‐up information was collected by telephone or outpatient records. Patients underwent chest CT scans every six months in the first two years postoperatively and annually thereafter. Information including recurrence and survival status, imaging results, medications, and post intervention status was recorded.

Recurrence was divided into three categories according to International Thymic Malignancy Interest Group (ITMIG) criteria.^1^ The postoperative effect of MG was examined by a skilled neurologist according to Myasthenia Gravis Foundation of America criteria defining post intervention status.^2^

### Statistical analysis

Statistical analyses were performed using SPSS version 19.0 statistical software. Normally distributed continuous data are presented as mean and standard deviation, while abnormally distributed data are presented as median and interquartile range. Categorical data are presented as frequencies and percentages. Survival curve and recurrence‐free curve were calculated using the Kaplan‐Meier method. A multivariate Cox regression analysis was performed to analyze prognostic factors of survival and recurrence‐free status. Uni‐ and multivariate logistic regression analyses were performed to analyze the predictive factors of postoperative myasthenic crisis (POMC). *P*‐values <0.05 were considered statistically significant.

## Results

### Demographic data

A total of 194 patients were included in this study. According to Osserman classification, there were 56 type I, 52 type IIa, 67 type IIb, 14 type III, and five type IV. None of the patients received chemotherapy or radiotherapy prior to surgery. However, 157 patients were treated preoperatively with pyridostigmine bromide (90–480 mg/day), five with pyridostigmine bromide and tacrolimus, and five with intravenous immunoglobulin; the other 27 patients received no medication due to mild symptoms. A total of 22 patients had other autoimmune diseases, including seven with thyroid diseases, 11 with skin‐related diseases (including leukoderma, eczema, and psoriasis), one with ulcerative colitis, one with polymyositis, one with ankylosing spondylitis, and one with rheumatoid arthritis. The demographic data are listed in Table 1.

**Table 1:** Demographic, perioperative, and pathological data

Variable	
Age (years), mean ± SD	49.9 ± 12.8
Sex, male/female	99/95
Diameter (cm), mean ± SD	4.4 ± 2.0
Surgical procedure, n (%)	
VATS	137 (70.6%)
Sternotomy	57 (29.4%)
Operation time (minutes), mean ± SD	136.6 ± 46.5
Blood loss (mL), mean ± SD	129.3 ± 287.4
Postoperative drainage (days), mean ± SD	3.3 ± 1.6
Length of postoperative stay (days), mean ± SD	8.3 ± 7.4
Osserman classification, n (%)	
I	56 (28.9%)
IIa	52 (26.8%)
IIb	67 (34.5%)
III	14 (7.2%)
IV	5 (2.6%)
Masaoka stage, n (%)	
I	58 (29.9%)
II	86 (44.3%)
III	35 (18.0%)
IVa	15 (7.7%)
World Health Organization classification, n (%)	
A	1 (0.52%)
AB	42 (21.6%)
B1	43 (22.2%)
B2	69 (35.6%)
B3	13 (6.70%)
Mixed B types	25 (12.9%)
MNT†	1 (0.52%)
Resection status, n (%)	
R0	184 (94.8%)
R1	7 (3.6%)
R2	3 (1.5%)
Perioperative complications, n	
Myasthenic crisis	20
Bleeding	2
Deep vein thrombosis	4
Cardiac events	6
Diaphragmatic paralysis	1
Pneumonia	1

† MNT, micronodular thymoma with lymphoid.

### Perioperative results and pathological data

VATS thymectomy was performed in 137 patients; two were converted to open thymectomy due to invasion of the great vessels. Transsternal thymectomy was performed in 57 patients. One patient was treated with mechanical ventilation due to POMC and died of ventilator‐associated pneumonia at the second month after VATS thymectomy. There were no other perioperative deaths. The perioperative and pathological data are listed in Table 1.

Perioperative data of the VATS and transsternal groups are shown in Table 2. VATS patients had a smaller tumor diameter, less blood loss, a shorter postoperative stay, and a lower rate of complications (*P* < 0.05). However, the transsternal group had more patients in the late stage and more patients with bulbar symptoms than the VATS group, which may have led to the differences in perioperative data.

**Table 2:** Comparison of clinical and perioperative data between VATS and transsternal group

Variable	VATS	Transsternal	*t*‐test or x^2^	*P*‐value
N	137	57		
Age (years), mean ± SD	50.8 ± 12.3	47.8 ± 13.7	1.526	0.129
Sex (male/female)	68/69	31/26	0.364	0.547
Diameter (cm)	3.8 ± 1.6	6.0 ± 2.0	−8.009	0.000
Operation time (min), mean ± SD	137.6 ± 44.3	134.3 ± 51.6	0.448	0.654
Blood loss (mL), mean ± SD	92.4 ± 194.6	217.4 ± 425.3	−2.805	0.006
Postoperative drainage (days), mean ± SD	3.1 ± 1.5	3.7 ± 1.9	−2.195	0.029
Postoperative stay (days), mean ± SD	6.7 ± 3.6	12.2 ± 11.7	−5.003	0.000
MG Osserman classification, n (%)				
I + IIa	85 (62.0%)	23 (40.4%)	7.676	0.006
IIb + III + IV	52 (38.0%)	34 (59.6%)		
Masaoka stage				
I + II	114 (83.2%)	30 (52.6%)	19.676	0.000
III + IVa	23 (16.8%)	27 (47.4%)		
WHO classification, n (%)				
A + AB+MNT	34 (24.8%)	9 (15.8%)	5.788	0.447
B‐types	103 (75.2%)	48 (84.2%)		
Complications	18 (13.1%)	18 (31.6%)	9.057	0.003

### Postoperative treatment and follow‐up results

A total of 86 patients received postoperative adjuvant therapy, of whom 77 underwent radiotherapy (two could not finish treatment due to radiation pneumonitis), six underwent chemotherapy, and three underwent radiotherapy and chemotherapy. Of the 194 patients, 170 (87.6%) were successfully followed up. The median follow‐up period was 45 months (range: 2–114 months).

**Figure 1:**
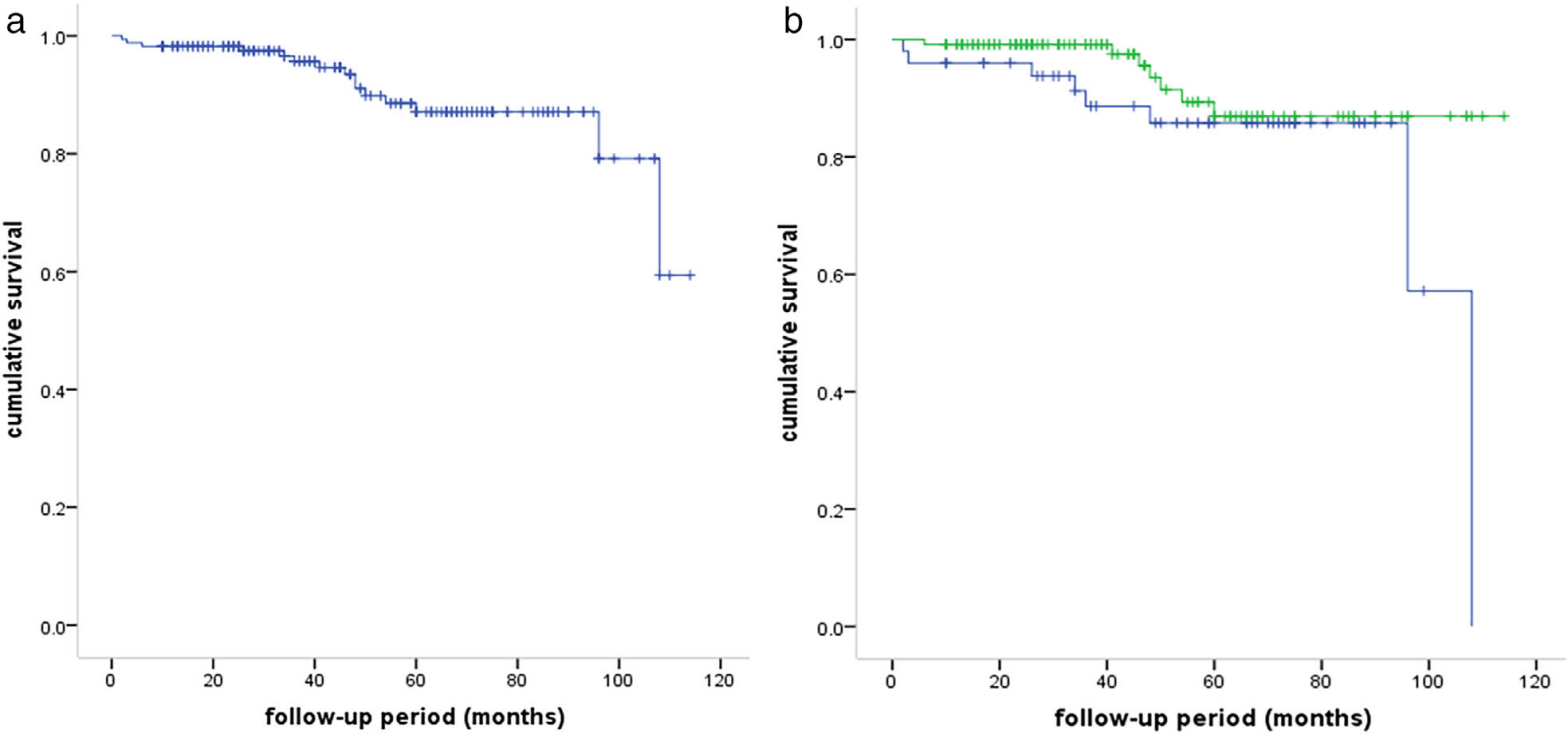
Survival curve of MG patients with thymoma. (**a**) Survival curve of whole group (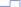) survival curve, and (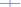) censored, and (**b**) Survival curve of two groups with different procedures (*P* = 0.109) surgical procedure, (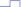) transsternal, (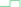) VATS, (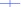) transsternal‐censored, and (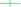) VATS‐censored.

For the whole group, the five‐year OS rate was 81.9%. The median recurrence‐free period was 39.5 months (range: 2–114 months). There were 16 recurrences among 170 patients (9.4%). According to ITMIG criteria, three patients had local recurrence, 10 had regional recurrence, and three had distal recurrence (two lung metastases, one chest wall metastasis). A total of 15 patients died: eight of MG, five of thymoma recurrence, one of cerebral infarction, and one unknown. The survival curves of the whole group and groups by different procedures are shown in Fig 1a,b.

### Risk factors for thymoma prognosis and recurrence

With regard to age, sex, Osserman classification, resection status, surgical procedure, Masaoka stage, complication, WHO type, postoperative therapy, and tumor recurrence as independent variables, the Cox regression analysis demonstrated that age, Masaoka stage, and recurrence were prognostic factors of OS (Table 3). Meanwhile, Cox regression analysis indicated that recurrence tended to occur in patients with Masaoka stage III + IV, while older patients had a lower risk of recurrence (Table 4).

**Table 3:** Cox regression analysis of risk factors for overall survival

Variable	HR	95% CI	*P*‐value
Age (continuous variable)	1.068	1.011–1.129	**0.019**
Sex			0.633
Female	Reference	—	
Male	0.733	0.205–2.624	
Osserman classification			0.636
I + IIa	Reference	—	
IIb + III + IV	1.37	0.372–5.043	
Resection status			0.468
R0 resection	Reference	—	
R1/2 resection	1.802	0.368–8.830	
Surgical procedure			0.586
Transsternum	Reference	—	
VATS	1.502	0.348–6.493	
Masaoka			**0.009**
I + II	Reference	—	
III + IV	8.584	1.717–42.907	
Complication			0.778
No	Reference	—	
Yes	1.261	0.252–6.304	
WHO type			0.565
A + AB+MNT	Reference	—	
B‐types	0.583	0.093–3.664	
Postoperative therapy			0.101
No	Reference	—	
Yes	0.186	0.025–1.386	
Tumor recurrence			**0.001**
No	Reference	—	
Yes	66.592	5.546–799.511	

*P*‐values < 0.05 are in bold font.

**Table 4:** Cox regression analysis of risk factors of tumor recurrence

Variable	HR	95% CI	*P*‐value
Age (continuous variable)	0.91	0.849–0.976	0.009
Sex			0.236
Female	Reference	—	
Male	2.682	0.524–13.725	
Osserman classification			0.569
I + IIa	Reference	—	
IIb + III + IV	1.529	0.355–6.580	
Resection status			0.811
R0 resection	Reference	—	
Non‐R0 resection	1.252	0.200–7.843	
Surgical procedure			0.698
Transsternum	Reference	—	
VATS	0.707	0.123–4.076	
Masaoka			**0.006**
I + II	Reference	—	
III + IV	13.126	2.066–83.408	
Complication			0.462
No	Reference	—	
Yes	0.47	0.063–3.520	
WHO type			0.97
A + AB+MNT	Reference	—	
B‐types	81 341.079	0.000–6.50E261	
Postoperative therapy			0.346
No	Reference	—	
Yes	3.728	0.241–57.575	

*P*‐values < 0.05 are in bold font.

### Risk factors of POMC

A total of 20 patients experienced POMC in the first month after surgery. Univariate logistic regression analysis indicated that bulbar symptoms (Osserman IIb + III + IV), surgical procedure, and blood loss were risk factors for POMC. Multivariable logistic regression analysis was conducted including six factors with a *P*‐value < 0.1 identified by univariable analysis. Only the presence of preoperative bulbar symptoms (Osserman IIb + III + IV) was indicated as an independent risk factor for POMC (Table 5).

**Table 5:** Univariable and multivariable analysis of risk factors of POMC

	Univariate model			Multivariate model		
Variables	OR	95% CI	*P*‐value	OR	95% CI	*P*‐value
Age	1.003	0.967–1.041	0.871	—	—	—
Sex	2.242	0.815–6.167	0.118	—	—	—
Osserman (IIb + III + IV)	8.000	2.247–28.477	**0.001**	6.541	1.732–24.698	**0.006**
Masaoka (III + IV)	2.536	0.954–6.743	0.062	0.986	0.298–3.259	0.982
WHO (B‐types)	5.864	0.760–45.224	0.09	2.994	0.364–24.598	0.307
Surgical procedure	3.026	1.158–7.908	**0.024**	0.533	0.182–1.566	0.253
Surgery time	1.008	0.999–1.017	0.087	1.006	0.994–1.017	0.338
Blood loss	1.001	1.000–1.002	**0.022**	1.001	0.999–1.002	0.358
Postoperative therapy	0.900	0.330–2.453	0.837	—	—	—
R0‐resection	1.025	0.123–8.558	0.982	—	—	—

*P*‐values < 0.05 are in bold font.

### Post intervention status of MG

**Figure 2:**
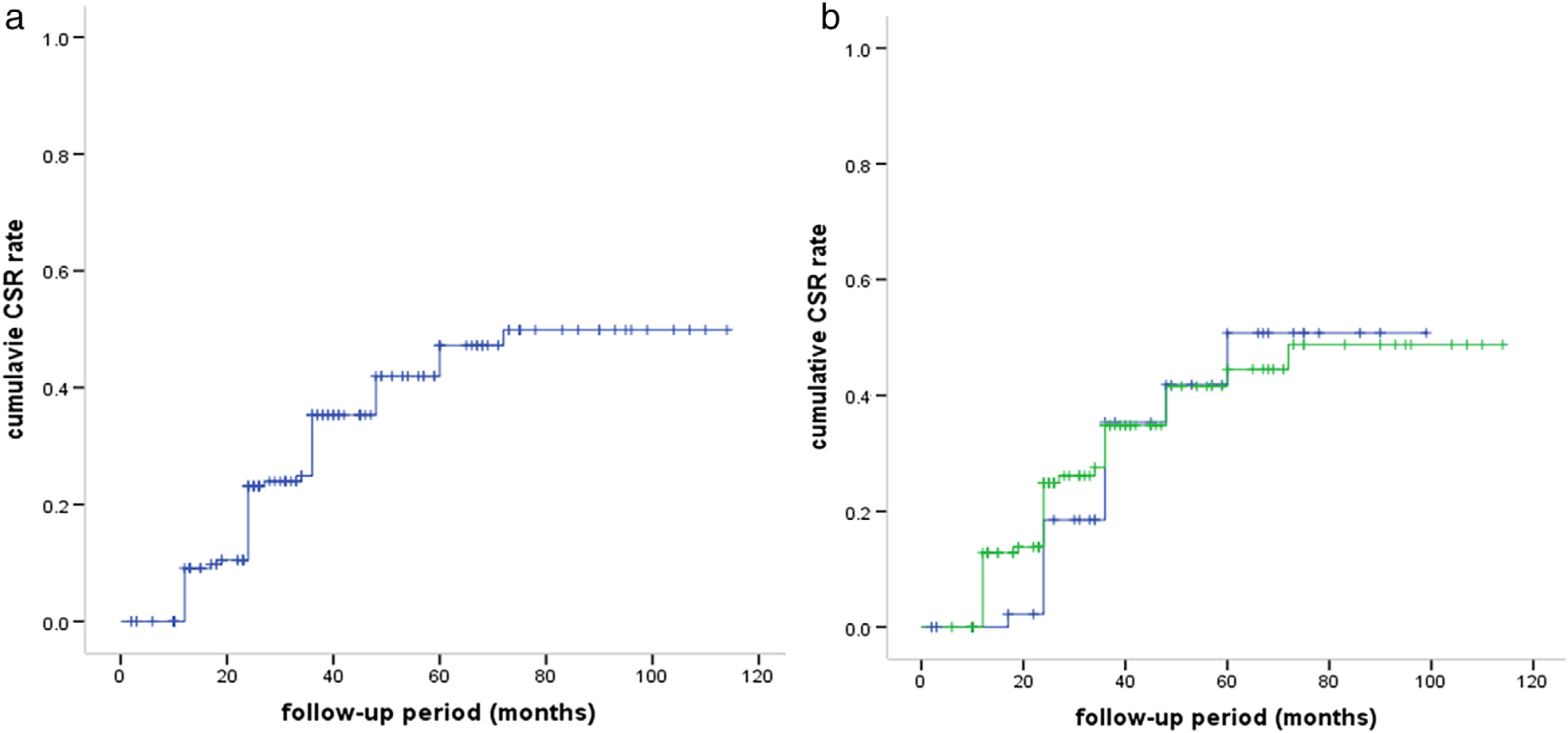
The cumulative CSR rate after surgical resection. (**a**) CSR of the whole group (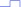) CSR curve, and (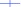) censored, and (**b**) Comparison of CSR rate between VATS group and transsternal group (*P* = 0.751) surgical procedure, (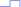) transsternal, (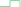) VATS, (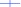) transsternal‐censored, and (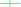) VATS‐censored.

Finally, 162 patients were included to evaluate the post intervention status of MG. A total of 55 patients (34.0%) achieved complete stable remission (CSR), three patients (1.85%) achieved pharmacologic remission (PR), 37 patients (22.8%) had minimal manifestations (MM), 42 patients (25.9%) had improved (I), nine patients (5.5%) were unchanged (U), four patients (2.5%) were worse (W), four patients (2.5%) had exacerbation (E), and eight patients (4.9%) died of MG (D). The overall effective rate, including CSR, PR, MM, and I, was 84.5% (137/162). The cumulative CSR rate was 19.3% at the end of the second postoperative year and rose to 44.1% in the fifth year (Fig 2). There was no significant difference in the CSR rate (32.7% vs. 36.7%, *P* = 0.622) and effect rate (84.1% vs. 79.6%, *P* = 0.248) between the VATS and transsternal groups. Multivariate Cox regression analysis indicated that older patients or thymomas of B types had a lower probability of achieving CSR (Table 6).

**Table 6:** Cox regression analysis of risk factors for CSR

Variable	HR	95% CI	*P*‐value
Age (continuous variable)	0.971	0.949–0.994	0.012
Sex			0.657
Female	Reference	—	
Male	1.136	0.648–1.989	
Osserman classification			0.382
I + IIa	Reference	—	
IIb + III + IV	0.765	0.419–1.396	
Resection status			0.554
R0 resection	Reference	—	
Non‐R0 resection	1.523	0.378–6.141	
Surgical procedure			0.422
Transsternum	Reference	—	
VATS	1.288	0.694–2.388	
Masaoka			0.664
I + II	Reference	—	
III + IV	0.823	0.343–1.978	
WHO type			**0.024**
A + AB+MNT	Reference	—	
B‐types	0.479	0.252–0.909	
Postoperative therapy			0.295
No	Reference	—	
Yes	1.392	0.749–2.584	

*P*‐values < 0.05 are in bold font.

## Discussion

An extended thymectomy is recommended for MG patients with thymoma, which means that not only the thymoma, but also the entire thymic tissue and anterior mediastinal fat tissue should be removed during surgery. Maximal excision of ectopic thymic tissue and microthymoma^3^ is considered important for MG treatment. At present, minimally invasive surgery for thymectomy is mainly through VATS, and sternotomy is recommended for thymomas of large size or invasion to great vessels. According to previous studies, compared with the open approach, VATS therapy had significantly less blood loss, less pain, shorter length of postoperative stay, and more cosmetic and similar long‐term prognosis of thymomas.^4^, ^5^, ^6^ To remove both the entire thymic tissue and anterior mediastinal fat tissue, VATS extended thymectomy can be employed with unilateral (right‐ or left‐sided), bilateral, subxiphoid,^7^ transcervical, or combined strategies. In this study, VATS accounted for 70.6% (137/194) of cases, and all were carried out using a unilateral approach (mainly right‐sided). The mean surgery duration was 137.6 minutes, the average postoperative stay was 6.7 days, and the complication rate was 5.8%, which were all similar with, or even better than, former studies.^4^ Therefore, VATS is a safe and effective surgical method for selected MG patients with thymoma.

Previous articles have reported that WHO classification, Masaoka stage, and R0 resection status may influence the recurrence and long‐term prognosis of thymomas.^8^, ^9^ COX regression analysis in our study indicated that Masaoka stage III + IV, not‐R0 resection, and recurrence were independent risk prognostic factors, but older age seemed to be a protective factor for recurrence, which was similar to the study by Li.^10^ The role of postoperative radiotherapy for thymomas remains controversial.^11^, ^12^ A previous study pointed out that adjuvant radiotherapy within one month after thymectomy may be helpful in controlling postoperative MG, such as decreasing the possibility of POMC and raising the probability of reaching CSR, but no prognostic benefit for thymoma was found.^13^ In our study, no significant influence of adjuvant radiotherapy on the prognosis of thymomas or postoperative effect of MG was indicated.

Thymomas are low‐grade malignant tumors, and the main treatment is surgery. One‐third of patients with thymoma have MG. The existence of MG complicates the treatment of thymoma and may have a negative effect on prognosis. Previous studies have indicated that MG is an indicator of poor prognosis for thymomas.^14^, ^15^ Worsened myasthenic symptoms or crisis may delay postoperative adjuvant treatment, reduce tolerance to radiotherapy or chemotherapy, and increase perioperative mortality. However, some experts indicated that MG may be a positive prognostic factor,^8^, ^16^ possibly because thymomas can be diagnosed earlier due to MG symptoms, which can increase the R0 resection rate and improve prognosis. In our cohort, a total of 15 patients died, eight of which (53.3%) died of MG aggravation or myasthenic crisis (MC) and five (33.3%) died of recurrence of thymoma. Yu *et al*.^14^ also reported that the main cause of death for thymoma patients with MG was myasthenic crisis.

MC is a life‐threatening medical emergency requiring early recognition and ventilatory support. POMC can be the main cause of death in thymoma patients with MG after thymectomy. MG must be fully assessed and treated effectively before surgery. The optical time for thymectomy is when symptoms of MG are light and stable, especially in patients with severe symptoms and rapid progression. Alternatively, POMC is quite likely to occur.^17^ A recent meta‐analysis^18^ identified the presence of preoperative bulbar symtoms as the most reliable preoperative risk of POMC, and a history of preoperative MC can increase the risk of POMC. Many other preoperative factors may be related to POMC, such as pulmonary function, immune suppressants, thymoma, and MG duration.^19^ Studies including only thymoma patients with MG indicated that WHO classification (B2 + B3) and not‐R0 resection may also be independent risk factors for POMC.^20^, ^21^ A total of 20 patients (10.3%) developed POMC in this study, and one died of ventilator‐associated pneumonia in the second month after surgery. We identified only the presence of bulbar symptoms (Osserman IIb + III + IV) as an independent risk factor of POMC, similar to most previous studies, but no other factors reached statistical difference.

The effective rate of thymectomy for treatment of MG was 75%–90%, the CSR rate was 16%–59.5% according to previous reports,^22^, ^23^, ^24^ and more than 10% of MG patients had a poor postoperative effect of MG, although they accepted systematic standard therapy for MG after surgery. In our study, the five‐year CSR rate was 44.1%, while the effective rate was 84.5%, similar to previous studies. Moreover, a few thymoma patients without MG reportedly developed MG after thymectomy, even after extended thymectomy.^25^, ^26^, ^27^ High‐level serum acetylcholine receptor antibodies may be a risk factor associated with the development of postoperative MG. These phenomena indicate that the pathogenesis of MG is a complicated process of immune imbalance, and perhaps thymoma was not the only cause and some other mechanisms were involved. Many factors could influence the postoperative effect of MG, such as age, sex, preoperative MG duration, and preoperative immunosuppressor. The existence of thymoma may be a negative factor for MG. However, a considerable difference was seen among different studies.^22^, ^28^, ^29^ In our study, older age and B‐type thymomas were independent negative factors for CSR. In contrast to other studies in which age was usually switched to a categorical variable, age was a continuous variable in our study.

Compared with previous studies, our study included only MG patients with thymoma and had a relatively larger sample size. We emphasize the importance of analyzing the postoperative effect of MG in these patients. However, several limitations should be emphasized. First, this study was retrospective, so some selection bias may have been present. Second, the follow‐up period was rather short, so the efficacy of thymectomy for thymomas may not have been sufficiently evaluated. Third, most patients were not graded according to Myasthenia Gravis Foundation of America classification. Therefore, we did not include it in the statistical analysis. Thus, a longer follow‐up period and patients with more detailed information are required in further studies.

In conclusion, extended thymectomy should be performed in patients with thymoma and MG. Age, Masaoka stage, and recurrence were prognostic factors for OS. The presence of bulbar symptoms was an independent risk factor for POMC. The VATS procedure could achieve similar treatment effects of MG with thymoma. Age and WHO classification may influence the postoperative effect of MG.

## Disclosure

No authors report any conflicts of interest.
